# Healthy Living with MCI: Implementation of a Statewide Education Program

**DOI:** 10.1002/alz70858_105531

**Published:** 2025-12-26

**Authors:** Dorothy F. Edwards, Jennifer McAlister, Bonnie L. Nuttkinson, Art Walaszek, Nathaniel A. Chin

**Affiliations:** ^1^ Wisconsin Alzheimer's Disease Research Center, University of Wisconsin School of Medicine and Public Health, Madison, WI, USA; ^2^ Wisconsin Alzhemer's Disease Research Center, Madison, WI, USA; ^3^ Achool of Medicine and Public Health, University of Wisconsin Madison, Madison, WI, USA; ^4^ University of Wisconsin‐Madison School of Medicine and Public Health, Madison, WI, USA; ^5^ Wisconsin Alzheimer's Disease Research Center, Madison, WI, USA; ^6^ University of Wisconsin‐Madison, Madison, WI, USA; ^7^ Alzheimer's Disease Research Center, University of Wisconsin‐Madison, Madison, WI, USA; ^8^ Department of Medicine, University of Wisconsin‐Madison School of Medicine and Public Health, Madison, WI, USA

## Abstract

**Background:**

Increasing understanding of MCI has been identified as a public health priority^1^, however MCI, as a concept, is difficult for professionals, patients, and the public to grasp^2^. There is a need for holistic, person‐centered education to support health and wellbeing of persons with MCI and their families^3^ (Mpofu 2024). In response to the need for community education the Wisconsin ADRC launched the Healthy Living with Mild Cognitive Impairment (HLMCI) education series to support persons with MCi and their care partners and promote health behaviors known to reduce dementia risk.

**Method:**

HLMCI is a quarterly series offering guidance and science‐backed strategies for living and coping with a diagnosis of MCI for communities across Wisconsin. Each session is led by the WADRC Medical Director and Clinical Core Co‐Leader together with a subject matter expert. Ample time for questions and answers are included in each event. Session topics are selected, guided by the Lancet Commission report on brain health risk factors with preference given to requests from program participants. The program is offered in 7 locations across the state. Including rural communities.

**Results:**

In 2024, the most popular topic was “You are More than Your Diagnosis” with 160 registrants. We averaged 112 registrants per session, 28% report a diagnosis of MCI, 35% were care partners and 35% were health or social service professionals. The majority of attendees are women, 45% are URGs. Although the median age of registrants was 55, the programs attract individuals ranging in age from under 35 to over 75; 52 % reported they were interested in finding out if they have amyloid or tau proteins in their brain. Approximately 40% attended 2 or more sessions last year. Satisfaction ratings of the topics and presenters are high ranging from 92 to 94 % across 2024 sessions.

**Outcome:**

Healthy Living with MCI is meeting an unmet need for information and guidance regarding lifestyle changes and activities to support brain health. While the program is designed to support individuals living with mild cognitive impairment, it also provides reliable and unbiased education and support to care partners and health professionals.

